# From integrative genomics to systems genetics in the rat to link genotypes to phenotypes

**DOI:** 10.1242/dmm.026104

**Published:** 2016-10-01

**Authors:** Aida Moreno-Moral, Enrico Petretto

**Affiliations:** Program in Cardiovascular and Metabolic Disorders, Duke-National University of Singapore (NUS) Medical School, Singapore

**Keywords:** Integrative genomics, Systems genetics, eQTL, Gene network, Rat

## Abstract

Complementary to traditional gene mapping approaches used to identify the hereditary components of complex diseases, integrative genomics and systems genetics have emerged as powerful strategies to decipher the key genetic drivers of molecular pathways that underlie disease. Broadly speaking, integrative genomics aims to link cellular-level traits (such as mRNA expression) to the genome to identify their genetic determinants. With the characterization of several cellular-level traits within the same system, the integrative genomics approach evolved into a more comprehensive study design, called systems genetics, which aims to unravel the complex biological networks and pathways involved in disease, and in turn map their genetic control points. The first fully integrated systems genetics study was carried out in rats, and the results, which revealed conserved *trans*-acting genetic regulation of a pro-inflammatory network relevant to type 1 diabetes, were translated to humans. Many studies using different organisms subsequently stemmed from this example. The aim of this Review is to describe the most recent advances in the fields of integrative genomics and systems genetics applied in the rat, with a focus on studies of complex diseases ranging from inflammatory to cardiometabolic disorders. We aim to provide the genetics community with a comprehensive insight into how the systems genetics approach came to life, starting from the first integrative genomics strategies [such as expression quantitative trait loci (eQTLs) mapping] and concluding with the most sophisticated gene network-based analyses in multiple systems and disease states. Although not limited to studies that have been directly translated to humans, we will focus particularly on the successful investigations in the rat that have led to primary discoveries of genes and pathways relevant to human disease.

## Abstract (precis)

**Summary:** This Review describes the most recent advances in the field of systems genetics, focusing on the rat. We cover from the first integrative genomics strategies (such as mapping of expression QTLs or eQTLs) and conclude with the most sophisticated gene network-based analyses in multiple systems and disease states.

## Introduction

Several gene mapping strategies such as genome-wide association studies (GWAS; see Box 1 for a glossary of terms), whole-exome sequencing (WES) and whole-genome sequencing (WGS) are now widely applied to study the genetic etiology of complex diseases (Altshuler et al., 2010). These approaches have led to the identification of thousands of genes, genetic variants and mutations that contribute to (or cause) human disease. However, this yield of genetic susceptibility data has not been mirrored by similar success in mapping the complex pathways and molecular interactions that underlie pathogenic processes. Too often the above-mentioned gene mapping strategies have been employed in conjunction with a reductionist study design, which, for the most part, is focused on the identification of single disease genes and mutations, essentially through analysis of one gene at a time. From a translational perspective, the identification of single genes (and mutations) predisposing to disease is hardly informative of the best point for therapeutic intervention, as this strategy ignores the cellular context in which genes operate and the role of other contributing factors (e.g. gene-by-environment interactions) in human disease (Romanoski et al., 2010). Moreover, when applied to human complex traits and disease, traditional genome-wide mapping approaches face significant power limitations because they require the assessment of large population samples – usually, more than 1000 individuals to detect major effect mutations or 10,000 individuals to detect common disease-risk variants (Hong and Park, 2012). Collecting data in large population samples is often hampered by technical hurdles involved in human phenotyping, e.g. difficulty of accurate monitoring of blood pressure in tens of thousands of individuals and inadequate access to tissues for analysis.

**Box 1. Glossary**

**Cellular-level traits:** quantitative traits measured at the level of the cell, usually assessed in homogeneous cell populations (e.g. an immortalized cell line) or in a tissue sample.

***Cis*-eQTL:** broadly speaking, this refers to local regulation of a gene. In humans, a gene is usually considered to be *cis*-regulated if there is a local genetic variant controlling the expression level of the gene. However, the distance between the genetic variant and the regulated gene does depend on factors such as genetic mapping resolution, linkage disequilibrium patterns, etc. In practice, a criteria used to define *cis*-eQTLs in humans is based on a *cis* window, which is commonly defined as ±1 Mbp around the transcript start site (TSS) of the regulated gene (Lonsdale et al., 2013).

**Clustered regularly interspaced short palindromic repeats (CRISPR)/Cas:** a genome-editing tool that allows the targeted disruption or replacement of the sequence of a gene(s) of choice.

**Co-expression network:** undirected network graph representing the co-expression relationships between sets of genes; this might be indicative of co-regulated genes in a given cell type or tissue.

**Comparative genomics:** a strategy based on the comparison of genomic features across different organisms, commonly used to translate findings from the rat (and other model organisms) to humans.

**Copy number variation (CNV):** a type of structural variant in which a DNA segment is present at a variable copy number in comparison with a reference genome.

**eQTL** **hotspot:** a genomic locus that regulates the expression levels of a large number of genes located elsewhere.

**Gene Ontology (GO):** defines concepts/classes used to describe gene function, and the relationships between these concepts. It classifies gene functions along three aspects: molecular function (molecular activities of gene products), cellular component (biological material, where gene products are active) and biological process (pathways and larger processes made up of the activities of multiple gene products).

**Genome-wide association studies (GWAS):** analysis of large sample cohorts to identify associations between common genetic variants and a disease trait.

**Master genetic regulator:** a gene harboring sequence variants that control a gene co-expression network.

**Principal component (PC) analysis:** a variable reduction procedure aimed to develop a smaller number of artificial variables (called principal components) that will account for most of the variance in the observed data. These principal components can then be used as ‘surrogate’ variables to summarize the main patterns of variability in the data (where the first component extracted accounts for a maximal amount of total variance in the observed variables).

**Quantitative trait locus (QTL):** a genomic locus that regulates a quantitative trait. Depending on the trait under genetic control, a QTL can be referred to as eQTL (regulation of gene expression), mQTL (regulation of metabolite), methQTL (regulation of methylation), histoneQTL (regulation of histone modification), miQTL (regulation of miRNA), pQTL (regulation of protein) or sQTL (regulation of splicing or relative transcripts abundances).

**Segregating population:** population with inter-individual genetic variability that can be generated by crossing two (or more) donor parents with different genetic backgrounds. Genetic variants that segregate in the population can be assessed by means of several genotyping techniques, which enable study of the co-segregation of genetic variants and phenotypic features in the population (Jansen, 2003).

**Single nucleotide polymorphism (SNP):** natural variation at the single nucleotide level in the DNA sequence, detected at the population level in a particular species.

***Trans*-eQTL:** refers to distal regulation of a gene. A gene is considered to be *trans*-regulated when its gene expression levels are influenced by a genetic variant located further away from the physical location of the gene itself. In humans, a practical criteria that is frequently used to define a *trans*-eQTL is a minimum distance of 1 Mbp between the genetic polymorphism and the transcript start site (TSS) of the regulated gene (Lonsdale et al., 2013).

**Transcription activator-like effector nuclease (TALEN):** a genome editing method that can be applied for the targeted disruption of one or more genes.

**Whole-body trait/phenotype:** any phenotype measured at the level of the whole organism, e.g. blood pressure, body mass index. This refers also to disease traits, e.g. hyperglycemia, hypertension and cardiovascular disease.

Despite the success of many gene mapping studies for several human diseases, a major unresolved issue concerns how the information encoded at the DNA level (e.g. genotypes) is translated to complex phenotypes and disease. Specifically, once DNA sequence variation is linked to whole-body phenotypes (Box 1) by GWAS, WES or WGS approaches, the question remains as to which molecular and signaling pathways are actually involved in the disease process under investigation, and which of these should be targeted to design new or better therapeutics. Therefore, genetic and biomedical research is now moving towards a comprehensive ‘systems-level’ description of the molecular processes underlying disease that goes beyond the identification of single genes and disease-predisposing mutations. In this case, the overall aim is to map pathogenic pathways, and to understand their regulation at the genetic and epigenetic levels. This shift towards systems-level analyses has been facilitated by the increasingly reduced costs and ongoing improvements of high-throughput ‘omics’ technologies – such as genomics, proteomics and metabolomics – which allow the simultaneous examination of thousands of genes, proteins and metabolites in a cost-effective way (Soon et al., 2013). The ‘omics’ revolution occurring in biology and biomedical research effectively enables the study of genes, their gene products (e.g. mRNA, proteins) and regulatory functions within multiple cell types and systems, providing unprecedented opportunities to understand the complex molecular basis of disease. Here, we start by delineating the integrative genomics approach and then summarize the recent progresses in systems genetics as an effective strategy to link genotypes to complex phenotypes and elucidate underlying biological processes, with a focus on applications in the rat for the study of complex disease.

## From integrative genomics to systems genetics

**Fig. 1.**
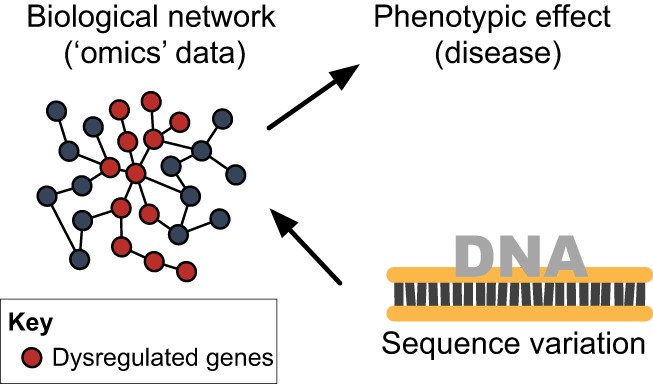
**Systems genetics overview.** Systems genetics aims to identify genetic variant(s) associated with biological networks that are dysregulated in disease to ultimately pinpoint ‘master genetic regulators’ (i.e. the primary drivers) of disease-associated processes.

One of the first strategies that emerged in the ‘omics’ era is integrative genomics, which refers to the *in silico* integration of different layers of ‘omics’ data, typically with the goal of identifying genetic variants that control specific cellular-level traits (such as DNA sequence variants regulating transcript abundance; Box 1) (Giallourakis et al., 2005; Ware et al., 2012). Systems genetics stemmed from this approach; however, it is a more comprehensive integrative strategy than integrative genomics. In contrast with integrative genomics, systems genetics aims to identify the major (genetic) determinants of disease and complex traits explicitly through the modeling and analysis of biological networks (Fig. 1). In a typical systems genetics study, DNA sequence variations are treated as the naturally occurring source of ‘genetic perturbation’ of the biological processes (represented as a biological network) that play a role in the development of disease. The genetic perturbation is sequentially linked to biological data, typically cellular-level phenotypes (e.g. RNA and protein expression within biological networks), and in turn associated with complex whole-body phenotypes and disease (e.g. blood pressure or obesity). As such, systems genetics goes beyond traditional integrative genomics by leveraging so-called ‘systems biology’ strategies based on the study of complex interactions within biological components (which include molecules, cells, tissues or organisms) to identify DNA variants together with biological molecular networks active in disease (Civelek and Lusis, 2013). The integration of different layers of ‘omics’ data also enables researchers to uncover the specific molecular interactions (e.g. protein-protein interactions or mRNA-mRNA co-expression) that are dysregulated in disease, and ultimately can lead to the identification of functional targets for therapeutic intervention (Rotival and Petretto, 2014; Yang et al., 2015). A systems genetics study typically requires the comprehensive molecular profiling (i.e. gene expression, protein and metabolite levels) of tissues and access to cellular systems relevant to disease pathogenesis using populations in which natural genetic variability between individuals has also been assessed. Although any population sample, family pedigree or cross of laboratory animals can be considered, the advantages of using segregating populations (Box 1) to link genetic variation to cellular-level traits has been highlighted (Jansen and Nap, 2001). DNA sequence variation is often assessed at the genome-wide level using single nucleotide polymorphism (SNP) arrays, a technology that allows the genotyping of millions of common genetic variants per sample in a single experimental run (LaFramboise, 2009) (Box 1).

**Fig. 2.**
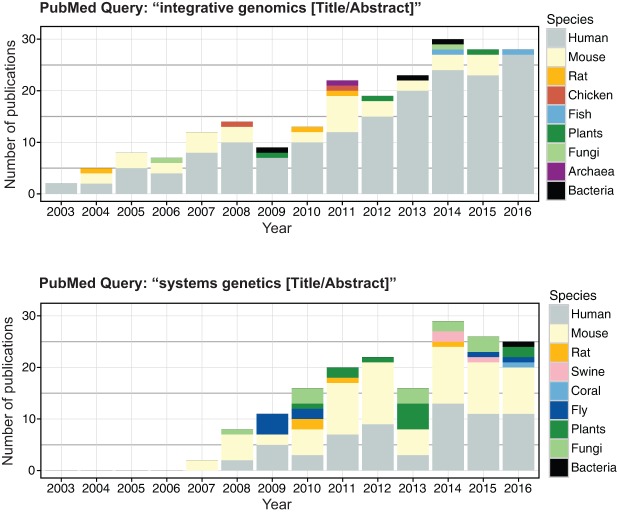
**Graph displaying the number of journal research articles obtained when querying PubMed for integrative genomics and systems genetics publications.** The results are broken down by year and species. PubMed was queried on the 14/07/2016. From the results, the publication type “Journal Article” was selected (references with the publication type “Review” were discarded). When the PubMed entries for the papers matching the queries did not include information about the model organism, this was added to the entry by manual curation of the paper. In addition, the results of the queries were also manually curated in order to remove review articles incorrectly annotated in the “Journal Article” category.

Several high-throughput techniques are used to measure molecular phenotypes and collect diverse ‘omics’ data. For instance, transcriptomics data is currently collected by next-generation sequencing (NGS) technologies such as RNA-sequencing (RNA-seq) (Wang et al., 2009), whereas mass-spectrometry and nuclear magnetic resonance (NMR) are often the methodologies of choice for proteomic (Boersema et al., 2015) and metabolomic (Fuhrer and Zamboni, 2015) data collection. As a direct result of the development of these widely accessible high-throughput techniques for molecular and phenotypic analyses, integrative genomics and systems genetics studies have grown in number and scope over the last decade. We queried the National Center for Biotechnology Information (NCBI) literature database, PubMed (http://www.ncbi.nlm.nih.gov/pubmed), to retrieve ‘integrative genomics’ and ‘systems genetics’ publications, and our search highlighted the growing use of these strategies in biomedical research in recent years (Fig. 2). Although humans are the predominant ‘model organism’ of choice, the earliest systems genetics studies (from 2007 and onwards) were primarily carried out in rodents (particularly in mice; Fig. 2). Overall, several different model organisms have been exploited for systems genetics studies because of their lower complexity compared with humans, ease of access to different cells and tissues, and reduced dimensionality of the ‘omics’ data collected (e.g. smaller genomes). These features facilitated the initial full-scale application of the systems genetics approach to the study of complex traits and human disease in model organisms.

## Data modeling strategies used in system genetics

There are two key types of genome-wide data modeling strategies that are used (and often integrated) in systems genetics studies: quantitative trait locus (QTL; Box 1) mapping of cellular-level traits, and gene regulatory network analysis. Each of these approaches has been extensively used in isolation in integrative genomics, for instance using expression QTL (eQTL) mapping (see below). Before discussing the full-scale systems genetics integration of these methods, we will give a brief overview of each data modeling strategy.

### QTL mapping

QTL mapping analysis focuses on finding statistical associations between genomic loci (e.g. DNA sequence variants such as SNPs) and quantitative variation in phenotypic traits. In the simplest case, a statistical test is applied to every genetic marker (e.g. SNP) and individual trait (e.g. body weight) assessed in a population sample. In this test, the hypothesis under testing is whether the sequence variant is affecting the trait, for instance whether the presence of a specific allele at a given genomic locus is associated with increased body weight in the population. QTL analysis can be carried out for any kind of measurable phenotypic trait, including cellular-level or tissue-level phenotypes. This gives rise to several types of QTL depending on the phenotypic trait under scrutiny that is associated with the genomic loci: eQTL refers to the association between a locus and gene expression levels, metabolite QTL (mQTL) refers to the association between a locus and metabolite levels, protein QTL (pQTL) refers to the association between a locus and protein levels, methylation QTL (methQTL) refers to the association between a locus and DNA methylation levels, histone QTL (histoneQTL) refers to the association between a locus and histone modification levels, microRNA QTL (miQTL) refers to the association between a locus and microRNA levels, and splicing QTL (sQTL) refers to the association between a locus and changes in relative abundance of transcript isoforms or presence of specific exons in transcripts. The resolution of QTL mapping is heavily dependent on the genetic crosses and number of informative genetic markers involved; often, QTL mapping identifies only large genomic regions associated with phenotypic traits in which several candidate genes could be equally implicated. Additional fine mapping strategies, for example using congenic mapping (Denmark et al., 2008), haplotype analysis and direct sequencing of candidate genes, are usually required to pinpoint the gene and locate the single nucleotide variant underlying the trait of interest.

Historically, eQTLs have been one of the first types of QTL identified for cellular-level phenotypes (Brem et al., 2002) and are now commonly used in integrative genomics and systems genetics studies. Since the first eQTL studies in the early 2000s – conducted in yeast – different terminologies have been introduced to classify eQTLs, including *cis-* and *trans-*acting eQTLs (Jansen and Nap, 2001) or local and distal eQTLs (Albert and Kruglyak, 2015). Thus, eQTLs can be classified as *cis-*acting when the expression of the gene maps to the gene itself; often, such variants act in an allele-specific manner [the expression of the copy of the gene located in the homologous chromosome is not affected by the genetic variant (Albert and Kruglyak, 2015)]. In the case of *trans-*acting eQTLs, the expression of the gene maps to other genomic locations (Jansen and Nap, 2001). *Trans*-regulated eQTL genes can arise as the result of different regulatory mechanisms of expression, including genetic variants located at regulatory regions (e.g. an enhancer) or variants that affect the function of a transcription factor (Zhu et al., 2008). Numerous studies have identified distinctive features of *cis*- and *trans*-eQTLs; for instance, *cis*-eQTLs often exert stronger effects on gene expression than *trans*-eQTLs, and *trans*-eQTLs appear to be more tissue-specific than *cis*-eQTLs (Box 1) (Petretto et al., 2006; Breitling et al., 2008; Fairfax et al., 2012). eQTL mapping has shown to be very valuable to annotate the functional context within which the many genetic variants identified by genome-wide mapping approaches operate. Thus, the integration of eQTLs and GWAS data can be informative to uncover molecular pathways in disease (Nica and Dermitzakis, 2013).

### Gene regulatory network and pathway analysis

Systems genetics is centered on the identification of biological networks (typically gene networks) and pathways involved in disease. The analysis of gene regulatory networks represents a key step of the systems genetics strategy, as it allows one to uncover genetic and cellular interactions associated with a particular pathological state: information that is not attainable by traditional single-gene mapping strategies (i.e. GWAS, WES and WGS). Generally speaking, a gene regulatory network consists of genes, which are represented by nodes in the network, and their functional relationships, represented by edges connecting the nodes. There are several techniques for gene regulatory network inference and analysis (reviewed in Rotival and Petretto, 2014). A type of network commonly considered in systems genetics studies is the gene co-expression network, where genes (nodes) are linked by undirected edges representing the inferred functional relationship between the connected genes in a given biological context (e.g. a particular tissue or developmental stage) (Box 1). Genes that are co-regulated and belong to the same regulatory network are likely to be detected as being co-expressed in the tissue or cell type where they operate (Mackay, 2014). These co-expressed genes can uncover the presence of a specific biological process (Martínez-Micaelo et al., 2016), which might be dysregulated in disease (e.g. inflammatory response revealed by co-expression of pro-inflammatory genes). Beyond the identification of known biological process, co-expression network analysis can be used to infer and refine the molecular function of genes with poor or unknown annotation. Using the ‘guilty by association’ principle, the co-expression of an uncharacterized gene with other genes of known function can help in defining the function of the former. For instance, gene co-expression analysis in the human heart allowed McDermott-Roe and colleagues to infer a previously unknown function for the enzyme endonuclease G in fundamental mitochondrial processes occurring in cardiac hypertrophy (McDermott-Roe et al., 2011). Gene regulatory networks can also be analyzed (and reconstructed) across different cell types or tissues and across multiple conditions (e.g. between cases and controls in human disease studies), which allows the detection of co-expressed genes, also called gene modules, that elicit differential (or common) activation of molecular processes (Tesson et al., 2010; Xiao et al., 2014).

The identification of a gene regulatory network is typically a first step in a systems genetics study. Once gene networks have been inferred in a given system, a wide range of approaches can be applied to annotate the function of the network and to assess enrichments for specific pathways, disease-susceptibility variants and infer common regulatory mechanisms. For instance, one can (i) identify specific biological processes represented in the networks (e.g. by Gene Ontology functional enrichment analysis of the genes forming the network) (Box 1), (ii) associate the networks with disease susceptibility, e.g. by testing for enrichment of disease-susceptibility variants (e.g. SNPs uncovered by GWAS) that map to gene sequences in the network, and (iii) examine whether the genes in the network have common regulatory mechanisms, for example by testing for enrichment of transcription factor binding sites within the promoter of the genes in the network (Johnson et al., 2015b). In addition to using genetic susceptibility data such as GWAS-inferred SNPs, gene networks can be associated with disease by summarizing the variability of the network [e.g. by principal component (PC) analysis; Box 1] and then correlating the major PCs with the trait or disease (Langfelder and Horvath, 2008). This strategy originated from the genome-wide correlation analysis of individual cellular-level phenotypes (e.g. mRNAs, proteins, metabolites) and whole-body quantitative phenotypes (e.g. body mass index). The approach is based on the assumption that ‘intermediate’ cellular-level phenotypes can be associated with variation in whole-body traits linked to disease (Passador-Gurgel et al., 2007). Although it has proven to be a valuable strategy to uncover molecular phenotypes associated with specific traits – for example, the expression of certain genes has been shown to correlate with variation in cardiac mass (Petretto et al., 2008) – additional functional analyses are still required to validate causal relationships (Mackay et al., 2009).

As in the case of eQTL mapping, gene networks can be linked to the genome to find genetic variants responsible for the co-regulation of the genes in the network – so-called ‘master genetic regulators’ (Box 1). Dissecting the genetic regulation of gene networks inferred in a target tissue can uncover genetic control points of processes that are deregulated in the context of disease, which might then be used to design new drugs for therapeutic interventions. Gene networks can be linked to the genome by individual eQTL mapping of the genes in the network, which can point to a common regulatory locus in the genome (Kang et al., 2014). It is also feasible to map the variability of the whole network to the genome, and thereby define a ‘network-QTL’. To this aim, one can summarize the gene network profile into a single variable through a ‘variable reduction’ technique (e.g. by using the PCs of the network) and then map the network profile using eQTL-based approaches (Johnson et al., 2015a). Alternatively, the expression profiles of all network genes can be jointly mapped to the genome, which can boost the power to detect the network-QTLs [e.g. by multivariate Bayesian modeling (Bottolo et al., 2011)]. More recent algorithmic developments also allow networks reconstructed from multiple datasets to be jointly mapped to the genome to identify network-QTLs across multiple conditions (Lewin et al., 2016).

## The value of the rat in studies of complex disease

In comparison to other well-characterized model organisms (such as zebrafish or *Drosophila*), the genetic and phenotypic features of rodents more closely resemble those of humans. The rat is a mammalian species with a long-established history in biomedical research, leading to its detailed phenotypic characterization. Although the mouse has been the primary model of choice for immunological phenotyping and gene-targeting studies (Jacob and Kwitek, 2002), the rat is arguably the best model for the study of cardiovascular and metabolic diseases (among other diseases; see Aitman et al., 2016) because it facilitates a more accurate analysis of clinical and cellular phenotypes in the cardiovascular system (Gauguier, 2016). For instance, traditional QTL mapping has been extremely successful in the rat: 109 QTLs have been mapped in the rat for ‘heart’ traits (compared to 29 in mouse and 14 in human) and 453 QTLs have been mapped in the rat for ‘blood pressure’ traits (compared to 40 in mouse and 77 in human) [source: Rat Genome Database (RGD), http://rgd.mcw.edu, accessed on 02/07/16]. This success in QTL detection for cardiovascular traits in the rat is likely due to the fact that cardiovascular physiology is easier to monitor in the rat than in mice. This can be explained by the bigger size of rats, which, for instance, facilitates blood pressure monitoring via implantable radio telemetry or performing surgical procedures in the heart. In addition, cardiovascular physiology data in the rat has been collected to an extent that is not yet replicated in the mouse (Iannaccone and Jacob, 2009).

A wealth of physiological and pharmacological data has been collected throughout the years in the rat (Jacob and Kwitek, 2002; Shimoyama et al., 2015). Several genes and disease-associated pathways identified in the rat have been found to be conserved in humans and have enhanced our understanding of the genetic contributions to human diseases (some relevant examples are presented in Tables 1 and 2). The rat offers a source of biological material from organs and primary cells that cannot be easily accessed in humans (Gauguier, 2016), therefore providing a direct means to investigate genes, pathways and multiple layers of functional regulation of disease. Particularly advantageous for systems genetics studies, orthogonal ‘omics’ datasets can be easily collected from the same rat model and across different systems (e.g. different tissue types), effectively enabling systems-level investigations of disease in this model organism.

**Table 1.**
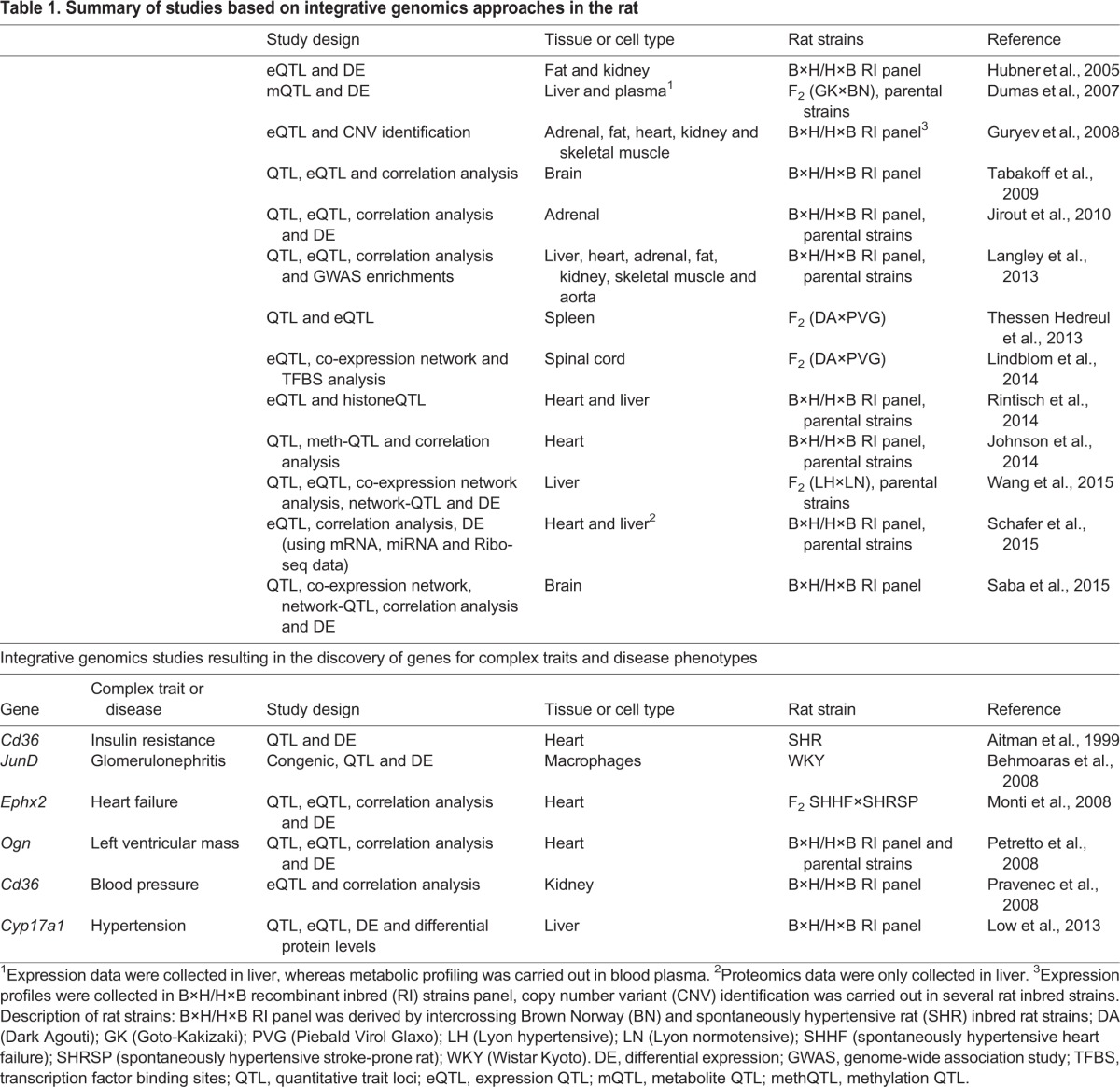
**Summary of studies based on integrative genomics approaches in the rat**

**Table 2.**
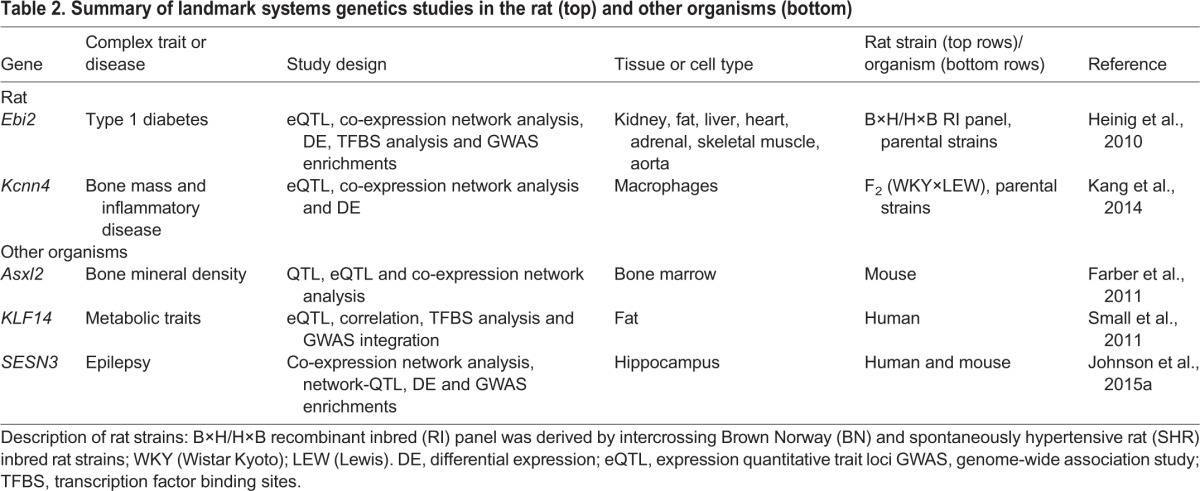
**Summary of landmark systems genetics studies in the rat (top) and other organisms (bottom)**

More recently, transgenic technologies have also been developed for application in the rat (i.e. nuclear cloning, lentiviral-mediated transgenesis, gene knockdown by RNA interference) (Jacob, 2010), and even the latest genetic technologies are ready to use. These include genome-editing technologies such as zinc-finger nucleases (Geurts et al., 2009; Cui et al., 2011), transcription activator-like effector nucleases (TALENs) (Mashimo et al., 2013; Ponce de León et al., 2014) and clustered regularly-interspaced short palindromic repeats (CRISPR)/Cas (Mashimo, 2014; Yoshimi et al., 2014) (Box 1). The recent availability of this wide range of genetic technologies bridges the gap between the rat model and other commonly used mammalian model organisms, notably the mouse (Aitman et al., 2008).

One of the key advantages of the rat model is the availability of specialized inbred strains and large panels for genetic mapping; so far, more than 500 inbred rat strains have been developed to model different human diseases (Aitman et al., 2008). The availability of rat genetic panels enables the mapping of cellular-level and whole-body phenotypes to the genome. Towards this goal, F_2_ crosses and panels of recombinant inbred (RI) strains have also been created. In particular, RI strains provide a valuable and cumulative source of data for genomic studies and can be fully characterized for genetic polymorphisms (Jirout et al., 2003). Among the genetic panels that have been developed for genetic research, the B×H/H×B rat RI strains [population generated by an original cross between the Brown Norway (BN) and the spontaneously hypertensive rat (SHR) (Pravenec et al., 1989)] has been the most extensively used genetic tool in systems genetics studies.

Since the publication of the draft sequence of the rat genome (Gibbs et al., 2004), further efforts have been made to improve the genomic resources available to the rat community (Atanur et al., 2013). Nowadays, it is feasible to systematically generate a wealth of phenotypic data (including cellular-level phenotypes) in the rat that can be integrated with available physiological, pharmacological and genomic information, to provide insight into human disease (Shimoyama et al., 2016). On the basis of these advantageous features and resources, the rat has been the most widely used model organism for integrative genomics and systems genetics studies of complex disease, as highlighted below.

## Landmark integrative genomics studies in the rat

**Fig. 3.**
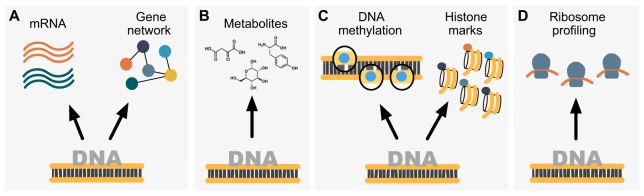
**Linking genotypes to cellular-level phenotypes in the rat.** (A) Many integrative genomics studies link gene expression (the transcriptome) to the genome (DNA sequence). All the examples discussed in the main text link gene expression to SNP markers, except in Guryev et al. (2008), in which gene expression is linked to CNVs. Studies linking mRNA levels to the genome in a single tissue include Monti et al. (2008), Petretto et al. (2008), Pravenec et al. (2008), Jirout et al. (2010), Low et al. (2013) and Thessen Hedreul et al. (2013), and across different tissues include Hubner et al. (2005) and Langley et al. (2013). Gene regulatory networks can also be linked to the genome (exemplified by Lindblom et al., 2014; Saba et al., 2015; Wang et al., 2015). (B) Metabolite levels, or the metabolome, can also be linked to the genome (exemplified by Dumas et al., 2007). (C) Epigenetic marks, including DNA-methylation levels (exemplified by Johnson et al., 2014) and histone marks (exemplified by Rintisch et al., 2014), can also be linked to the genome. (D) The ribosome profile, or translatome, can also be linked to the genome (as in Schafer et al., 2015).

This section aims to provide an overview of the main integrative genomics studies conducted in the rat, in which ‘omics’ data have been linked to the genome. A streamlined summary of studies is reported in Table 1 and the implemented approaches are displayed in Fig. 3.

### Linking the transcriptome to the genome: single- and multiple-tissue eQTL studies

Hubner et al. (2005) carried out the first multi-tissue and large-scale integrative genomics study in the rat model system. In this study, the authors assessed expression profiles in fat and kidney tissues in the B×H/H×B panel of rat RI strains (Table 1). They first investigated genes that are differentially expressed between the parental progenitor strains (BN and SHR) in both tissues, and then carried out genome-wide eQTL mapping, which identified thousands of *cis-* and *trans-*regulated genes in the rat. They provided a list of common and tissue-specific eQTLs and uncovered a preponderance of *trans*-acting eQTLs among the tissue-specific eQTLs, an observation that was further reinforced by subsequent studies across seven tissues (Petretto et al., 2006). In addition, Hubner and colleagues identified the regulatory genetic variants underlying the observed transcript differences for the most statistically significant *cis*-acting eQTL genes, and analyzed the colocalization of eQTLs with whole-body pathophysiological QTLs previously mapped in SHR. To obtain candidate genes for the regulation of human hypertension, they focused on eQTLs that colocalize with blood pressure-related QTLs in the rat (retrieved from the RGD). Using a comparative genomics analysis with humans (Box 1), i.e. by comparing the rat eQTLs with the human loci previously identified as human blood pressure-associated QTLs, the authors provided new insights into the contribution of genetic variants regulating gene expression in relevant target tissues to the molecular basis of human hypertension. The study also emphasized the potential of the B×H/H×B RI strains panel for the study of the genetics of any pathophysiological phenotype that segregates in this rat population (Hubner et al., 2005). Since the publication of this study, many other eQTL mapping investigations followed, with the overall aim to link the transcriptome to the genome in the rat model system.

An elegant example of integrative genomics applied to study neurobehavioral traits in the rat is the work performed by Tabakoff et al. (2009) (Table 1). In this study, the authors analyzed brain gene expression in the B×H/H×B panel of rat RI strains and measured alcohol consumption (a whole-body phenotype) to identify candidate genes that predispose to increased alcohol intake. They carried out correlation analysis between brain gene-expression levels and alcohol consumption, QTL analysis of alcohol consumption, and eQTL analysis in the rat brain. By integrating all results, the authors were able to identify genes that correlate with alcohol consumption and are also *cis*- and *trans*-regulated by the genomic variants located within the implicated QTL regions, thus pinpointing key genes underlying this whole-body trait in rats. The results were translated to humans by analyzing data from two human populations (Tabakoff et al., 2009). Follow-up studies provided additional functional validation supporting the role of the GABAergic system in alcohol consumption and other ethanol-related behaviors (Cruz et al., 2011; Saba et al., 2011). Another single-tissue eQTL study involved analysis of adrenal gland expression data in the B×H/H×B panel of rat RI strains, in order to identify sequence variations that influence catecholamine biosynthesis and storage (Jirout et al., 2010) (Table 1). In this study, the authors carried out differential expression analysis between the progenitor strains (again BN and SHR), correlation analysis between expression levels and biochemical phenotypes, QTL mapping of biochemical phenotypes and eQTL analysis in the B×H/H×B RI strains. They also overlapped the results with previously annotated QTLs, ultimately identifying primary genetic mechanisms for the regulation of hereditary hypertension (Jirout et al., 2010).

Another example of comparative genomics of rat eQTLs and human loci for hypertension was provided by Langley et al. (Table 1). Their aim was to explore the functional and regulatory mechanisms mediating the effects of genes previously reported to be associated with elevated blood pressure in 15 human GWAS (Langley et al., 2013). The results of this analysis supported the hypothesis that *trans*-eQTLs are conserved between rats and humans, and that they could represent the intermediate genes that connect GWAS SNPs with associated complex phenotypes (Fehrmann et al., 2011; Parks et al., 2013; Albert and Kruglyak, 2015). Moreover, Langley et al. suggested that the study of *trans*-eQTLs could contribute to the identification of the functional relevance and molecular pathways underlying the effects of genetic susceptibility variants identified in GWAS. Following this, similar strategies have been proposed by Björkegren et al. (2015) to unlock the heritability and genetic etiology of coronary artery disease using GWAS data. As a final example, a study published by Thessen Hedreul et al. (Table 1) involved the integration of eQTL and autoimmune encephalomyelitis QTL analysis, using gene expression profiles collected from spleens of an F_2_ population of rats. This approach identified several candidate genes and pathways involved in the regulation of autoimmune encephalomyelitis (Thessen Hedreul et al., 2013). In summary, these integrative genomics studies demonstrate how genome-wide expression analysis in pathophysiologically relevant tissues can be integrated with genetic variation data to identify *cis*- and *trans*-eQTLs, which in turn can pinpoint the genetic mechanisms underlying QTLs or GWAS loci associated with complex diseases. Beyond the widespread contribution of common genetic variants to regulate gene expression levels, there are other sources of inter-individual genetic variability that can have important functional effects on the transcriptome; these will be discussed below.

### Linking the transcriptome to the genome: the role of copy number variation

The rat has also been shown to be a valuable resource for the study of structural variants, such as copy number variants (CNVs; Box 1), that underlie complex physiological traits and disease. The utility of the rat model for the identification of CNVs relevant to complex human diseases was first demonstrated by Aitman and colleagues, who revealed that copy number polymorphism in the *FCGR3* gene (*Fcgr3* in rats) – which encodes low affinity immunoglobulin gamma Fc region receptor III – predisposes to glomerulonephritis in both rats and humans (Aitman et al., 2006).

A second example of a rat-based study of CNVs was provided by Guryev et al. (2008), who provided the first large catalog of functional CNVs in the rat (Table 1). The authors used both computational and experimental procedures to characterize CNVs in several widely used rat strains. They uncovered thousands of CNVs genome-wide, and found that these were non-randomly distributed across the rat genome; for example, they reported the tendency for CNVs to be present adjacent to telomeres and centromeres. Similar to the conservation of rat eQTLs with human disease loci (Hubner et al., 2005; Langley et al., 2013), these authors reported that many of the identified CNVs had been previously associated with human genetic disorders. For instance, among the 113 one-to-one orthologous genes that overlapped CNV regions in both rat and human, 80 genes were also listed in the Online Mendelian Inheritance in Man (OMIM) database (https://omim.org), including the transcriptional activator and repressor GLIS family zinc finger 3 (*GLIS3*), in which partial gene deletions cause neonatal diabetes and congenital hypothyroidism (Dimitri et al., 2011). In addition, the team integrated expression data from SHR, BN and the H×B/B×H RI strains panel in five tissues (adrenal, fat, heart, kidney and skeletal muscle) and computed the list of genes differentially expressed between BN and SHR for each tissue, which showed that ∼50% of the genes located within CNV regions were differentially expressed. In the case of the B×H/H×B RI strains panel, they showed that CNVs that regulate gene expression were mainly tissue-independent, and that some of the transcripts located within 0.5 Mb of a CNV were *cis*-acting eQTLs in all five tissues tested (Guryev et al., 2008). This study highlighted that, as in humans, CNVs play a pervasive role in the regulation of gene expression and disease in the rat. The conservation of rat and human disease-causing CNVs suggests the possibility of detailed functional studies in the rat model (Aitman et al., 2006), in particular in the case of human diseases for which fresh target-tissue samples are difficult to retrieve (e.g. heart or brain).

### Linking the transcriptome to the genome: gene co-expression network studies

Gene co-expression network analysis has also proven to be a valuable strategy to understand the regulation of gene expression relevant to human diseases. For instance, Lindblom et al. published a study in 2014 (Table 1) in which they performed eQTL mapping in data collected from spinal cord tissue of an F_2_ rat intercross (Lindblom et al., 2014). The aim of this work was to better understand the mechanisms regulating complement activation in the central nervous system. The authors identified clusters of *trans*-regulated genes that included several complement proteins of interest. This uncovered gene co-expression networks and functional processes involved in complement activation. Furthermore, by carrying out enrichments of transcription factor binding sites within the genes forming part of the *trans*-regulated networks, they identified a previously unknown FOX family transcription factor as a potential candidate for the regulation of a *trans*-eQTL cluster that includes the complement protein C3 gene (Lindblom et al., 2014). An elegant study by Saba et al. (Table 1) used the B×H/H×B RI strains panel to build co-expression networks in the brain and associate these to predisposition towards alcohol consumption (Saba et al., 2015). The integration of co-expression networks, network-QTL, correlation analysis, alcohol consumption QTL and differential expression analysis allowed the identification of common functional pathways relevant to this trait (Saba et al., 2015). Another example of integrative genomics in action, provided by Wang and colleagues, leveraged gene co-expression network analysis to shed light on metabolic syndrome in the Lyon hypertensive rat (Table 1). The team collected data on genotypes, 23 whole-body physiological traits and gene expression (using RNA-seq) from the liver of rats following an F_2_ intercross (Wang et al., 2015). Whole-body physiological traits QTL, eQTL and differential expression analyses were performed and integrated to identify genes involved in pathogenesis (by prioritizing differentially expressed eQTL genes that were located within a QTL for metabolic syndrome-related traits, as previously proposed by Morrissey et al., 2011). In addition, Wang and colleagues reconstructed gene co-expression networks, mapped these to the rat genome and searched for colocalization of metabolic syndrome-related QTL traits and network-QTLs, which revealed candidate genes underlying the intricate pathogenesis of metabolic syndrome. Among these genes, the authors identified the rat gene *RGD1562963* (an ortholog of the human gene *C6Orf52*) as a putative master regulator of a *trans*-eQTL network associated with body weight and blood pressure. Additional experimental validation of the role of *RGD1562963* in liver metabolism and metabolic syndrome has been planned by the authors (Wang et al., 2015).

### Linking the metabolome to the genome

Current high-resolution metabolomics technologies enable the comprehensive metabolic phenotyping of large cohorts of animal models and human populations, and these data can be integrated with genetic mapping analyses. mQTL studies in genetic mapping populations is a feasible strategy for the characterization of the multilevel control of metabolite abundance, which contributes to a deeper understanding of genome–phenotype relationships in disease processes (Gauguier, 2016). A landmark study published by Dumas et al. (Fig. 3B and Table 1) demonstrated the applicability of exploratory spectroscopic phenotyping analysis in the rat to link metabolite variation to the genome (Dumas et al., 2007). They used untargeted NMR spectroscopic analysis in plasma and genotype data to identify mQTLs from a rat cross and congenic strains models of diabetes. In doing so, for a set of ∼150 mQTLs the authors identified candidate metabolites that were regulated by these loci. As mQTLs can shed light on the biomarkers associated with diabetes, they also looked at colocalisation of mQTLs with previously reported diabetes-linked metabolic and physiological QTLs in the rat, thereby uncovering another level of biological complexity in this disease (Dumas et al., 2007). Similar studies of mQTLs have been carried out using mice, which identified genetically determined metabolites that could be candidate biomarkers for cardiometabolic syndrome (Cazier et al., 2012; Ghazalpour et al., 2014).

### Linking epigenetic marks to the genome

Epigenetics plays an essential role in the global regulation of gene expression and biological processes. Epigenetic marks can be linked to the genome because they provide information with respect to modifications of the DNA and associated proteins that is not captured by analysis of the primary DNA sequence alone (Rintisch et al., 2014). Post-translational modification of histones plays an important part in genome organization and regulation of gene expression. Rintisch et al. carried out a study (Fig. 3C and Table 1) in which they generated a histone modification map of the rat genome, and assessed to what extent patterns of histone modification are affected by DNA variation (Rintisch et al., 2014). The histone modification map was generated using chromatin immunoprecipitation (ChIP)-seq data of histone post-translational modifications in heart and liver tissues of BN, SHR and the B×H/H×B RI strains. They looked at four well-characterized histone methylation marks: H3K4me3, H3K4me1, H4K20me1 and H3K27me3 (in the case of the RI strains, they only looked at H3K4me3 and H3K27me3). Furthermore, they collected gene expression profiles and combined these with the histone ChIP-seq data. The authors analyzed the tissue specificity of histone-modification marks and mapped the histone marks H3K4me3 (associated with active promoters) and H3K27me3 (associated with gene silencing) to SNPs in the rat genome to identify *cis*- and *trans*-acting histoneQTLs. Then they investigated whether the identified histoneQTLs were also eQTLs and found that combining histone modification data with gene expression analysis boosted the power of eQTL detection, which represents a novel approach for the identification of genotype–phenotype relationships. Notably, the authors estimated that ∼36% of genes had a genotype-dependent effect on histone modifications that was not due to transcription. The results of this integrative genomics analysis provided evidence that histoneQTLs can predict gene expression levels and that joint analysis of histone modifications and gene expression data enhances the prediction of eQTLs in the rat (Rintisch et al., 2014). Because eQTLs in the rat can be used to make sense of human GWAS associations and elucidate the functional role of variants associated with disease susceptibility (Hubner et al., 2005; Langley et al., 2013), defining more eQTLs in disease-relevant tissues (which are more accessible in rats than in humans) can aid the identification of genetic mechanisms underlying human disease.

Another integrative genomics study focused on epigenetics was conducted by Johnson and colleagues (Fig. 3C and Table 1). In this study, the authors performed an integrative analysis of genotypes, phenotype and genome-wide characterization of cytosine methylation at the single nucleotide resolution in the rat (Johnson et al., 2014). Specifically, whole-genome bisulfite sequencing in the heart of SHR and BN rats was performed, and cytosine methylation levels at CpG dinucleotides were linked to pathophysiological phenotypes measured in the same animals. They identified differentially methylated sites in cardiac tissue between SHR and BN rats and carried out methQTL analysis in the B×H/H×B RI strains. This analysis revealed that most of the differentially methylated loci were *cis*-regulated and that the genetic regulation of DNA methylation in the rat heart was, for the most part, independent of cell type. In addition, they performed an association analysis in which they correlated variation in CpG methylation with 241 physiological quantitative traits measured across the B×H/H×B RI strains panel. Finally, they investigated the individual genetic variants responsible for the *cis*-regulatory control (including allele-specific methylation) observed in the analysis of methQTLs. This last analysis yielded an unexpected concordance between differentially methylated sites in the BN/SHR parental strains and allele-specific methylation, suggesting that CpG cytosine methylation is mainly *cis*-regulated by local sequence variations. Overall, this study illustrated that CpG methylation levels are under genetic control, providing a framework for the study of the molecular mechanisms underlying these processes, while also contributing to the understanding of the interrelation between the regulation of CpG methylation and pathophysiological cardiac phenotypes and biomarkers in the rat (Johnson et al., 2014). For instance, the team reported significant correlation between a locus-specific CpG methylation change in cardiac tissue and levels of serum chromogranin B, which is a correlate of sympathetic nervous system overactivity and has been proposed as a biomarker for heart failure (Røsjø et al., 2010).

### Linking the translatome to the genome

Schafer et al. recently pioneered a technique that allows genome-wide translation efficiency to be measured, and they used the method to study the role of translational regulation in complex traits in the rat model (Schafer et al., 2015). In their analysis, the authors performed RNA-seq and sequencing of ribosome-protected RNA fragments (Ribo-seq) in the heart and liver of SHR and BN inbred rat strains (Fig. 3D and Table 1). Using these Ribo-seq data, they were able to detect more disease-specific pathways as compared with the results of traditional differential expression analysis based only on RNA-seq. Integration of mass-spectrometry-based proteomics data in the liver confirmed that, at least in this tissue, Ribo-seq was a better proxy for estimating protein levels than RNA-seq. Additional integration of liver and heart eQTL data in the H×B/B×H RI strains panel revealed that ∼80% of the genes with an eQTL were also differentially translated between the two parental strains, suggesting that translation and transcription are correlated in many instances. However, the authors pinpointed several differentially translated genes with no overlap with any eQTL. This shows that Ribo-seq can identify genomic variants associated with differential translation that are missed by RNA-seq- or microarray-based eQTL studies. In addition, they noticed a higher density of SNPs in the 3′UTR of genes with translational control and, by additional integration of miRNA sequencing in the liver and heart, they found that differentially transcribed miRNAs are enriched in the 3′UTR of genes with differential translation. This elegant study integrates for the first time in the rat an additional layer of regulatory information (i.e. the translatome) with DNA sequence variation data, proposing Ribo-seq to be considered alongside RNA-seq analysis in future integrative studies that use this model system (Schafer et al., 2015).

## Integrative genomics studies that led to disease-gene identification

A pioneer example of integrative genomics study in the rat that led to the discovery of a gene underlying the regulation of complex disease was performed by Aitman and colleagues (Aitman et al., 1999). This work sought to identify candidate genes that underlie insulin resistance, defective fatty acid metabolism and hypertriglyceridemia in the SHR background (Table 1). First, the authors carried out QTL analysis for the traits insulin-mediated glucose uptake and catecholamine-mediated lipolysis. Second, by integrating the QTL data with gene expression profiling, they found that platelet glycoprotein 4 (*Cd36*) was located within the identified QTL region and differentially expressed between SHR and BN rats. Further characterization, including overexpression experiments in a transgenic mouse, established the role of *Cd36* as a regulator of whole-body lipid homeostasis, suggesting a role in the pathogenesis of insulin resistance syndromes (Aitman et al., 1999). Their findings were validated in an independent transgenic rescue study, in which transgenic expression of *Cd36* in SHR contributed to improved insulin resistance and lower levels of serum fatty acids (Pravenec et al., 2001). Following this, Pravenec et al. published a study in which they used integrative genomics based on eQTL analysis in the rat kidney to demonstrate a role for *Cd36* as a major determinant of blood pressure and risk for hypertension (Pravenec et al., 2008). Specifically, the authors combined eQTL analysis in the kidney of H×B/B×H RI strains with genome-wide correlation analysis between all *cis*-regulated QTLs in kidney and radio-telemetry measures of blood pressure (Table 1). *Cd36* yielded the strongest correlation with blood pressure variation. Additional experiments in transgenic SHR and congenic strains involving renal transplantation of kidneys with different versions of *Cd36* provided supporting evidence to the hypothesis that loss of *Cd36* leads to higher blood pressure levels (Pravenec et al., 2008).

Another rat study, from Behmoaras and colleagues (Table 1), combined congenic, linkage and genome-wide gene expression analyses to discover the activator protein-1 (AP-1) transcription factor JunD as a major determinant of macrophage activity and its association with glomerulonephritis susceptibility. Notably, this integrative genomics study revealed conservation of JunD function in macrophage activation between rats and humans, therefore suggesting a new therapeutic strategy for diseases characterized by inflammation and macrophage activation (Behmoaras et al., 2008).

Starting from an eQTL approach, a study by Monti et al. identified bifunctional epoxide hydrolase 2 (*Ephx2*) as a heart failure susceptibility gene (Monti et al., 2008) (Table 1). The authors combined invasive hemodynamic measurements, expression profiling and genome mapping in F_2_ hybrids bred from the spontaneously hypertensive heart failure-prone and the spontaneously hypertensive stroke-prone rats (the latter strain does not develop heart failure). Also in this study, eQTL analysis led to the initial identification of *Ephx2* as a strong *cis*-regulated gene located within a heart failure QTL in the rat. Furthermore, *Ephx2* was strongly correlated with ejection fraction (a clinical measure to characterize heart failure) and differentially expressed between the parental strains, an observation that was also validated at the protein level. Finally, the team showed that knockout of *Ephx2* in mice provides protection from cardiac arrhythmias and improvement of heart failure-related clinical parameters compared to wild-type mice (Monti et al., 2008).

A successful discovery that stemmed from genome-wide eQTL analysis in the rat relates to the osteoglycin (*Ogn*) gene, which our group identified as a major regulator of left ventricular mass (LVM) via the transforming growth factor beta (TGFβ) signaling pathway (Petretto et al., 2008). In this work, spearheaded by the Cook and Petretto laboratories at Imperial College London, we integrated eQTLs mapping in cardiac tissue with QTL analysis of LVM, by means of genome-wide correlation analysis of cardiac gene expression with LVM (Table 1), using the B×H/H×B RI strains. This approach resulted in the identification of an LVM QTL region in which *Ogn* was the strongest *cis*-regulated eQTL gene correlating with LVM and that was also differentially expressed between BN and SHR parental rat strains. Additional genome-wide correlation analysis of cardiac expression and LVM in a human cohort yielded *OGN* (as well as several members of the TGFβ pathway) as the gene most strongly associated with LVM. We also showed that an *Ogn* knockout mouse model displays lower LVM *in vivo*, providing compelling evidence for *Ogn* as a major regulator of LVM in rats, mice and humans (Petretto et al., 2008).

A study published by Low et al. involved the integration of transcriptome and proteome data, which led to the identification of the steroid 17-alpha-hydroxylase/17,20 lyase (*Cyp17a1*) as a candidate for blood pressure regulation (Low et al., 2013). The authors combined RNA-seq with liquid chromatography and mass spectrometry to analyze the transcriptome and proteome of liver tissue in two rat strains (BN and SHR). Comparison of the differentially expressed genes with the differential protein levels led to the identification of *Cyp17a1* as the most downregulated gene in SHR compared to BN at both the transcript and protein level (Table 1). Further analyses revealed that *Cyp17a1* is a *cis*-regulated gene, located within a blood pressure QTL, in the H×B/B×H RI strains. The authors identified a single nucleotide variant in the promoter of the gene as the most likely source of its variation in gene expression. The human ortholog, *CYP17A1*, had previously been found to be a top association hit in a blood pressure GWAS, further highlighting the power of the rat system to identify genes with a conserved role in human disease (Low et al., 2013).

## Landmark systems genetics studies in the rat

**Fig. 4.**
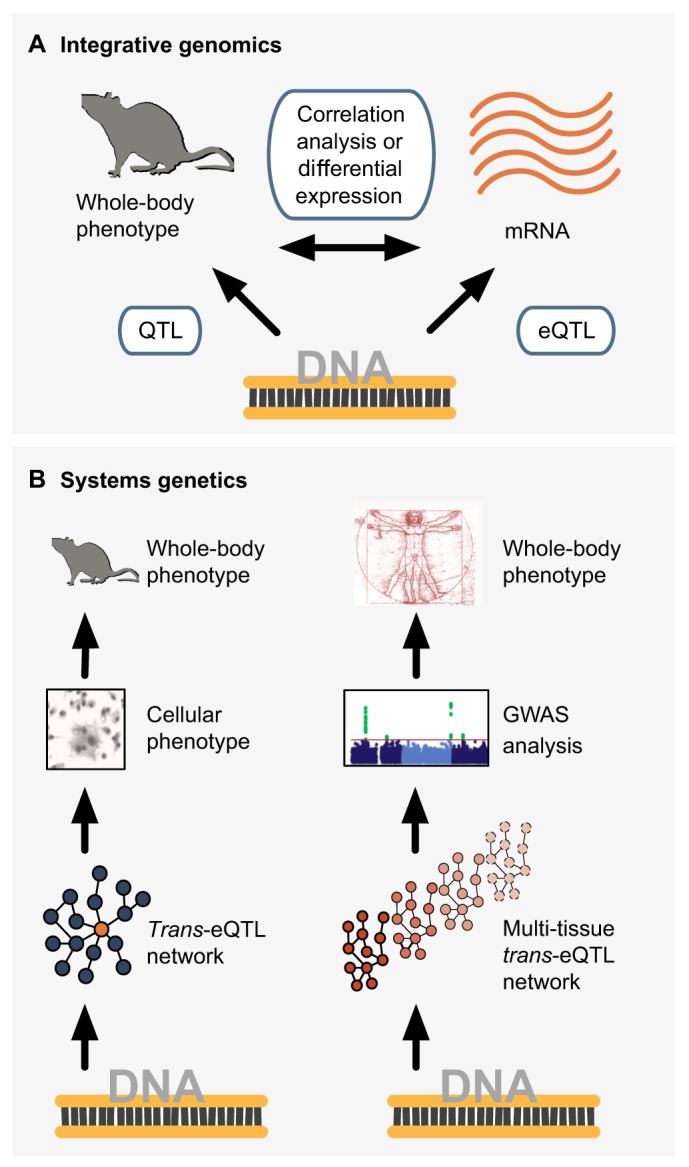
**Integrative genomics and systems genetics strategies in the rat.** (A) Integrative genomics studies have led to the identification of disease genes, for instance by integrating QTL data (Aitman et al., 1999; Behmoaras et al., 2008) or eQTL data (Monti et al., 2008; Petretto et al., 2008; Pravenec et al., 2008; Low et al., 2013) with analysis of gene expression in disease-relevant tissues and cell types. (B) Systems genetics studies have identified master genetic regulators of *trans*-eQTL networks. Left: *trans*-eQTL networks were associated with cellular-level traits driving whole-body phenotypes in rodents (i.e. bone homeostasis and inflammatory disease) (Kang et al., 2014). Right: *trans*-eQTL networks identified in the rat were conserved to humans and enriched for GWAS variants, therefore linking the networks to a whole-body disease phenotype (i.e. type 1 diabetes) (Heinig et al., 2010) (see Table 2).

In this section we present a summary of systems genetics analyses in the rat, with a focus on those studies where both master genetic regulators and genetic networks underlying complex disease have been identified and translated to humans (Table 2 and Fig. 4). Although we focus on the two main systems genetics studies carried out so far in the rat system (Heinig et al., 2010; Kang et al., 2014), we will also comment on related systems genetics investigations carried out in other species (humans in particular).

The first integrated systems genetics study was performed in the rat and the results were translated to humans (Heinig et al., 2010) (Fig. 4B, Table 2). In this work, integration of a transcription-factor-driven co-expression network with network-QTL mapping pinpointed the Epstein–Barr virus induced gene 2 (*EBI2*) as a master genetic regulator of a network of antiviral expression associated with type 1 diabetes (T1D) risk. Under the hypothesis that loci associated with perturbation of genetic networks could be relevant for disease risk, Heinig and colleagues carried out genome-wide co-expression network analysis in seven tissues from the H×B/B×H RI strains (liver, heart, adrenal, fat, kidney, skeletal muscle and aorta). This analysis led to the discovery of a co-expression network with an overrepresentation of genes involved in inflammatory processes, containing several targets of the transcription factor interferon regulatory factor 7 (*Irf7*). Bayesian network-QTL mapping (Petretto et al., 2010) in the seven rat tissues revealed that the genes in the network were controlled by a common regulatory hotspot (Box 1) that was located at the rat *Ebi2* gene locus. The authors also used comparative genomics to translate the findings to humans. These studies uncovered that the rat antiviral co-expression network is conserved in human monocytes (an observation that was replicated in two independent population cohorts) and that the human *EBI2* gene colocalizes with a T1D susceptibility locus previously identified by GWAS but not followed up (Wellcome Trust Case Control Consortium., 2007; Barrett et al., 2009). The human gene network was linked to susceptibility to disease: the authors demonstrated that the genetic sequences of the human genes included in the co-expression network were overrepresented for T1D GWAS susceptibility variants (Heinig et al., 2010). The genetic control of the network was also identified in human monocytes. However, this study highlights the power of the rat system for mapping the genetic control of complex regulatory networks to discrete genomic loci, which, when analyzed in humans, can reveal new determinants of complex disease or, as in this case, can be critical to functionally annotate and corroborate a previously reported GWAS signal for T1D.

A second system genetics study in the rat provides an example of how the characterization of clusters of *trans*-eQTLs can lead to the identification of *trans*-acting genetic regulators of complex traits (Fig. 4B, Table 2). In this example, Kang et al. performed genome-wide eQTL mapping in multinucleating macrophages in the rat [in a segregating population derived from Lewis and Wistar Kyoto inbred rat, an experimental model of glomerulonephritis (Behmoaras et al., 2008)]. This genome-wide eQTL analysis uncovered a large cluster of *trans*-eQTLs, which yielded the discovery of a gene co-expression network enriched for genes involved in macrophage multinucleation (termed ‘macrophage multinucleation network’). This network-QTL (coinciding with the single *trans*-eQTL hotspot) was located within the Trem (triggering receptor expressed on myeloid cells) gene family (Kang et al., 2014). Further functional analyses, together with siRNA-mediated knockdown of *TREM2* in both rat and human macrophages, evidenced that TREM2 was *trans*-regulating the macrophage multinucleation network in both organisms. Additionally, by using two independent murine models, the authors found that the most strongly *trans*-regulated gene of the network, *Kcnn4*, which encodes intermediate conductance calcium-activated potassium channel protein 4, is implicated in the regulation of macrophage multinucleation, bone homeostasis, inflammatory arthritis and glomerulonephritis. These findings provided novel insights into the molecular mechanisms of macrophage multinucleation, uncovered new regulators of these processes and offered novel therapeutic targets for inflammatory disease (Kang et al., 2014). This study highlights how the identification of *trans*-eQTL genes can lead to the discovery of networks (in this case underlying macrophage multinucleation) and genes regulating specific cellular processes with global phenotypic effects, as shown for two inflammatory diseases.

As well as these two rat studies, it is worth mentioning other notable and successful examples of system genetics studies performed in other animals. For example, using mice, Farber et al. identified *Asxl2* (additional sex combs like-2) as the gene underlying a bone mineral density (BMD) GWAS hit (Farber et al., 2011) (Table 2). In this study, eQTL and co-expression network analyses were carried out with data collected from a large mouse genetic panel. The mouse results were translated and integrated with human BMD GWAS data. Moving on to work carried out entirely in humans, a study performed by Small et al. (Table 2) identified Krüppel-like factor 14 (*KLF14*) as a *trans*-acting master genetic regulator of a gene network that regulates metabolic traits (Small et al., 2011). In this case, the genes in the network were mapping in *trans* (in human fat tissue) to the *KLF14* locus, which had been previously identified in GWAS studies as being associated with type 2 diabetes and levels of high-density lipoprotein cholesterol (Teslovich et al., 2010; Voight et al., 2010). Another human study, performed by Johnson et al., identified the sestrin-3 (*SESN3*) gene as a positive (*trans*-acting) master genetic regulator of an epileptic-gene network (Table 2). The gene network was reconstructed using genome-wide expression analysis of surgically resected hippocampi from individuals with temporal lobe epilepsy. The epileptic-gene network was associated with susceptibility to epilepsy by integration with separately obtained GWAS data. Finally, by providing experimental evidence in two model organisms – mouse and zebrafish – the authors demonstrated the cross-species conservation of the epileptic-gene network and the role of *SESN3* as a modulator of chemically induced seizures *in vivo* (Johnson et al., 2015a). These studies demonstrate how systems genetics can be applied to shed light on the molecular mechanisms underlying GWAS hits as well as to annotate the function of the many susceptibility variants identified for human disease.

## Concluding remarks

The plethora of disease-predisposing gene variants identified by GWAS, WES and WGS has not been paralleled by a similarly fast and far-reaching characterization of the associated biological processes and pathways. We foresee that the increased availability of ‘omics’ data generated across many model organisms, as well as in humans, is likely to yield a more powerful and synergistic platform for developing cross-species systems genetics approaches. Indeed, all successful systems genetics studies discussed in this Review took advantage of genome-scale datasets available in multiple species (rats, mice, humans, zebrafish, and others). In the near future, the increasingly common use of emerging genomics technologies, such as single cell sequencing (Tang et al., 2009) or chromosome conformation capture-based techniques (Jin et al., 2013), will provide more detailed genomic and regulatory information, which can be similarly integrated in more advanced systems genetics investigations. The rat, as well as other model organisms, will continue to be highly relevant for this upcoming stream of advanced system genetics studies of complex disease.

We believe that the attainment of several systems genetics studies in the rat has been greatly facilitated by substantial collaborative efforts of the rat genetics community, which for instance in Europe has been supported by more than 10 years of continued funding (e.g. the STAR, EURATools and EURATRANS projects). This enabled the rat genetics community to focus on the accumulation of extensive ‘omics’ and physiological phenotype data in the same genetic mapping populations [e.g. in the H×B/B×H recombinant inbred strains (Pravenec et al., 1989) or in outbred rats (Rat Genome Sequencing and Mapping Consortium et al., 2013)]. This strategic vision relied on the added value of measuring high-resolution phenotype data in the same system, which has been rewarding even when pursued in relatively small genetic crosses or mapping populations (e.g. in the 30 available H×B/B×H recombinant inbred strains). A similarly comprehensive and high-resolution collection of ‘omics’ and phenotypic measurements in relevant tissues and cell types is still not feasible in large human population cohorts. This unique wealth of ‘omics’, physiological and pharmacological data collected in the last 100 years in the rat makes this model organism an optimal system to apply integrative genomics and systems genetics to boost the discovery and functional characterization of genes and pathways for human disease.
